# Collagen9a1c localizes to collagen fibers called actinotrichia in zebrafish fins

**DOI:** 10.17912/micropub.biology.000747

**Published:** 2023-04-07

**Authors:** Hibiki Nakagawa, Toshihiro Aramaki, Shigeru Kondo, Junpei Kuroda

**Affiliations:** 1 Graduate school of Frontier Biosciences, Osaka University, 1-3 Yamadaoka, Suita, Osaka 565-0871, Japan

## Abstract

Teleost fish fins are supported by spear-shaped collagen crystals called actinotrichia. Actinotrichia are distributed radially at the distal end of the fins and thought to be necessary for proper formation of the fin and fin-bones. We previously reported that *collagen9a1c* ( *col9a1c* ) gene product is essential for the regular arrangement of actinotrichia using *col9a1c* -knockout zebrafish. Here, we examined the localization pattern of the EGFP-tagged Col9a1c protein in the fins to understand its role in the arrangement of actinotrichia. We found that EGFP-Col9a1c specifically localizes to actinotrichia.

**Figure 1. EGFP-Col9a1c localizes to collagen fibers called actinotrichia in caudal fin of zebrafish:**
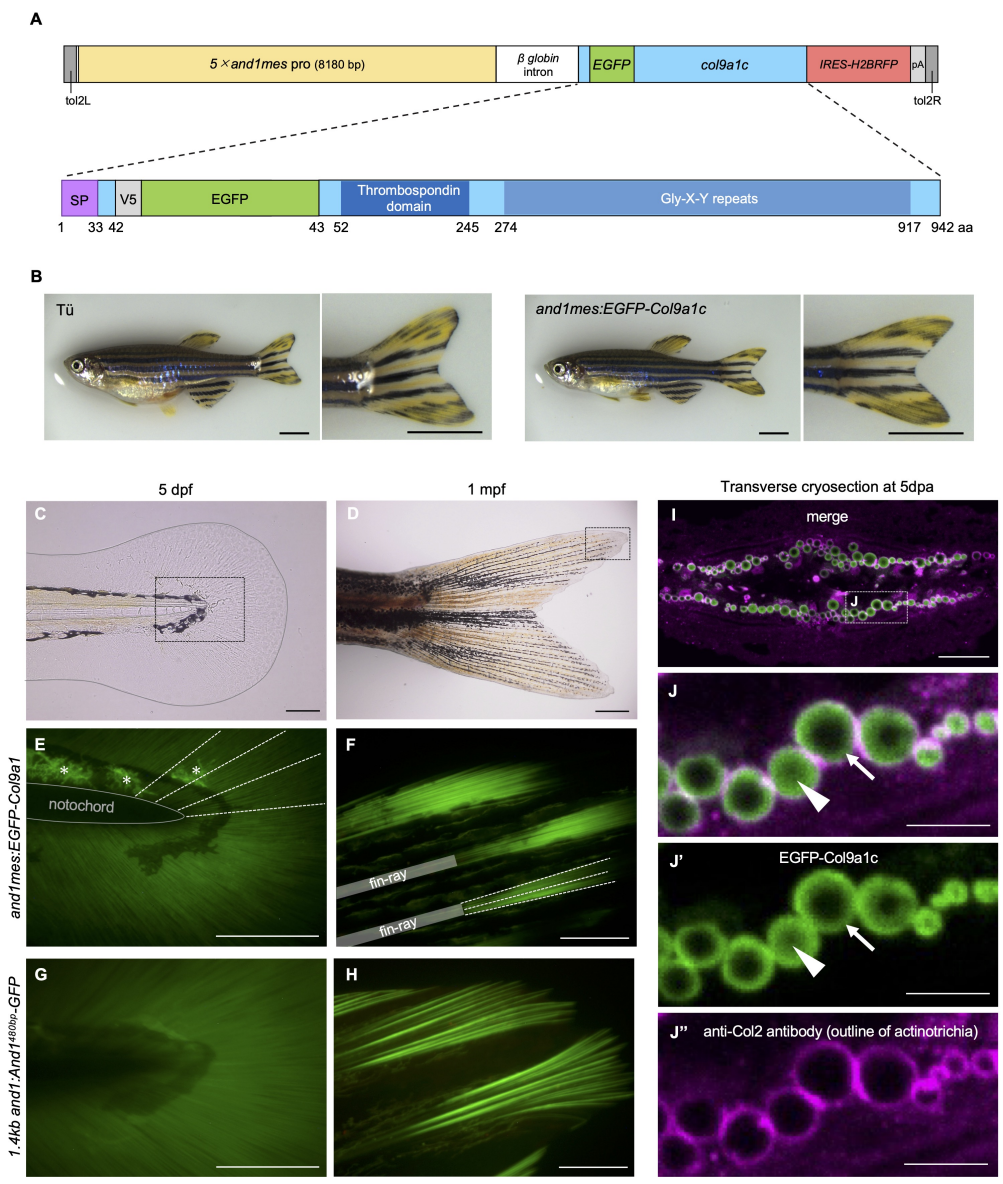
(A) Schematic illustration of the Col9a1c expression construct. The upper diagram shows the expression of EGFP-Col9a1c driven by the *actinodin1* promoter that is specifically active only in the mesenchymal cells ( *and1mes* pro, designed by Kuroda et al. (2020) based on the previous paper reported by Lalonde et al. (2016)), the lower diagram shows the EGFP-fusion Col9a1c. EGFP and V5 epitope tag were inserted between the 42nd and 43rd amino acids of wild-type Col9a1c. IRES-Hsa.HIST1H2BJ-mRFP (referred to as IRES-H2BRFP in this paper) was used to confirm that the introduced gene is expressed in the fins. (B) The phenotype of the transgenic zebrafish expressing an EGFP-Col9a1c fusion protein (Tg line name, ou2046Tg). The transgenic fish does not show any abnormalities in the shape of the body or the caudal fin. (C, D) Bright-field image of the caudal fin at 5 dpf (C) and 1 mpf (D). The grey line in C indicates the outline of the fin. The areas enclosed by black dotted lines in C and D represent near the center of the caudal fin and in the tip of the fin, respectively, and correspond to the observation areas of E and G, and F and H, described below. (E, F) Fluorescent images of EGFP-Col9a1c at 5 dpf (E) and 1 mpf (F). White dot lines indicate representative locations where the fluorescent signal was detected. The fluorescence indicated by the asterisks in E is the autofluorescence of xanthophores, not the Tg-specific signal. (G, H) Fluorescent images of actinotrichia visualized by And1-GFP (Tg line name, ou2106Tg (Kuroda et al., 2018)) at 5 dpf (G) and 1 mpf (H). At each stage, the localization of both EGFP-Col9a1c and And1-GFP is restricted to actinotrichia. (I) The transverse section of cryosection in the tip of an adult regenerating fin at 5 days post-amputation (dpa). Immunofluorescence staining for Collagen2 (magenta) was performed prior to cryosectioning to label the outline of actinotrichia in *and1mes:EGFP-Col9a1c* fish (EGFP-Col9a1c; green). (J–J’’­) Magnified image of the white dashed box in I. The fluorescence signals of EGFP-Col9a1c are specifically detected in actinotrichia. White arrows and arrowheads in J and J’ indicate the localization of EGFP-Col9a1c around actinotrichia surface and the inner center of actinotrichia, respectively. Scale bars: 3 mm in B; 100 µm in C­, E–H; 500 µm in D; 20 µm in I; 5 µm in J­–J’’.

## Description

In vertebrates, the body shape and its movement depend on the morphology and arrangement of each bone. Therefore, bones must be formed in the proper morphology and assembled in the proper arrangement. Fins of teleost are supported by straight fin-bones (fin-rays) that elongate from proximal to distal part of fins by adding bone-segments distally. At the distal tip of fin where the fin-rays are not yet formed, spear-shaped collagen crystals called actinotrichia provide physical support to fins. In the fin fold of embryo to larval fish, actinotrichia are radially and uniformly distributed. Then, as fin-rays are forming, actinotrichia gradually disappear from the proximal part of the fin and become located only at the distal tip of the fin-rays.

Many previous studies have shown that actinotrichia are essential structure for fin formation in development and also regeneration (Santamaría and Becerra, 1991; Quint et al., 2002; Zhang et al., 2010; Durán et al., 2011; Pfefferli and Jaźwińska, 2015; Bhadra and Iovine, 2015; Lalonde and Akimenko, 2018; König et al., 2018). Furthermore, we have previously reported that the *collagen9a1c* ( *col9a1c* )-knockout zebrafish shows disorderly arrangement of actinotrichia, resulting in dorsoventrally contracted caudal fin and curved, barely branched fin-rays (Nakagawa et al., 2022) . However, it is unclear how *col9a1c* contributes to the alignment of actinotrichia, which is essential for accurate fin and fin-ray formation. Therefore, in this report we investigate the localization of the *col9a1c* gene product by generating a transgenic zebrafish, which is the first step towards understanding its role in the arrangement of actinotrichia.

To express fluorescently labeled Col9a1c in zebrafish fins, EGFP was fused to the N-terminus of the mature Col9a1c protein (lower image in Fig. 1A and see Methods for details ). Since the promoter region of *col9a1c* was not identified in this study, we instead focused on the *actinodin1* ( *and1* ) promoter. It is reported that And1 is a component ECM protein of the actinotrichia (Zhang et al., 2010; König et al., 2018). In addition, Kuroda and colleagues found that the promoter of *and1* can be used for the fluorescent visualization of the actinotrichia in zebrafish fins (Kuroda et al., 2018; Kuroda et al., 2020) . Comparing the mRNA expression patterns of *and1* and *col9a1c* , both are strongly expressed in the mesenchymal cell region of the fin tip ( Fig. 1 and 2 in König et al., 2018; Fig. 1 in Nakagawa et al ., 2022). Based on the result, we decided to drive EGFP-Col9a1c with the mesenchymal cell-specific *actinodin1* promoter ( *and1mes* pro, upper image in Fig. 1A ) designed by Kuroda et al. (2020). This promoter was created based on the one previously reported by Lalonde et al. (2016). They confirmed that a small region from 1117­–967 bp of the 1941 bp genomic fragment of *and1* is specifically expressed in ectodermal cells. They therefore removed this 150 bp region and named the promoter *2PΔepi* , which is expressed specifically in mesenchymal cells. Kuroda et al. (2020) used this *2PΔepi* promoter with sequences including up to intron 1 as the *and1mes* promoter. In addition, upstream of this, a fragment of *2PΔepi* without the 200­–1 *and1* fragment (promoter region) was connected in four replicates to enhance expression. Together, we refer to them as *5×and1mes* promoter in this paper.

The transgenic zebrafish line, *5×and1:EGFP-col9a1c-IRES-Hsa.HIST1H2BJ-mRFP* (named ou2046Tg, referred to as *and1mes:EGFP-Col9a1c* in this paper), grew normally and were morphologically indistinguishable from the wild-type ( Fig. 1B ­). At 5 days post-fertilization (dpf), EGFP-Col9a1c were radially distributed in the entire of caudal fins. Then, at 1 month post-fertilization (mpf), the fluorescent signals of EGFP-Col9a1c were restricted at the tip of fin-rays (shown as white dot lines in Fig. 1E and F ). In the fins of *Tg* ( *1.4kb and1:And1 ^480bp^ -GFP* , named ou2106Tg) (Kuroda et al., 2018) , actinotrichia were specifically labelled with And1-GFP at both stage of 5 dpf and 1 mpf ( Fig. 1G and H ). We found that the distribution pattern of EGFP-Col9a1c in the *Tg* ( *and1mes:EGFP-Col9a1c* , ou2046Tg) was similar with the And1-GFP in the *Tg* ( *1.4kb and1:And1 ^480bp^ -GFP* , ou2106Tg) ( Fig. 1E ­­–H), indicating that Col9a1c can be localized to actinotrichia under the control of at least the *and1mes* promoter. We next observed the fluorescence of EGFP-Col9a1c in the cryosections of the adult regenerating fin to confirm the localization pattern of Col9a1c in more detail. The outline of the actinotrichia was labeled by anti-Collagen2 (anti-Col2) staining prior to cryosectioning. As shown in the Fig. 1I ­–J’’, the fluorescence of EGFP-Col9a1c was exclusively detected in the actinotrichia and not in other extracellular regions. In most actinotrichia sections, EGFP-Col9a1c proteins were accumulated near the surface of actinotrichia (white arrows in Fig. 1J, J ’), but in some actinotrichia sections, their distribution was extended to the inner center of actinotrichia (white arrowheads in Fig. 1 J, J ’). These findings suggest that Col9a1c is specifically localized in actinotrichia together with fibrillar collagens and actinodin family proteins. Note that it is not yet clear whether the construct ( *pTol2-5×and1mes:EGFP-Col9a1c-IRES-H2BRFP* ) is functional, as rescue assays have not been performed.

Previous studies have shown that actinotrichia are held by mesenchymal cells (MCs) (Wood and Thorogood, 1987; Böckelmann and Bechara, 2009; Kuroda et al., 2020) and Lalonde and Akimenko (2018) demonstrated that actinotrichia could not align in parallel in zebrafish pectoral and median fin under the condition of MCs ablation. In addition, Kuroda et al. (2020) recently showed that MCs use the physical force generated by the actin cytoskeleton to orient actinotrichia. Furthermore, it has also been shown that Collagen 9 generally act as an anchor between Collagen 2, a component of collagen fibers, and other extra cellular matrices (ECMs) (Eyre et al., 2004; Zaucke and Grässel, 2009; Firner et al., 2017). Together with the results of these studies, our findings suggest that Col9a1c may directly or indirectly (e.g., by binding other ECM components) intermediate the interaction between actinotrichia and MCs. To clarify this, future work will focus on investigating *in vivo* interactions between actinotrichia and MCs.

## Methods

**Zebrafish husbandry**

All experiments in this study were conducted in accordance with the guidelines and approved protocols for animal care and use at Osaka University, Japan. Zebrafish were maintained under standard laboratory conditions at 28.5 ℃ and a 14/10 h light/dark cycle. Live zebrafish in the experiments were used after anesthetizing with tricaine (MS-222).

**Transgenic zebrafish for visualizing Col9a1c protein**

The full-length *collagen9a1c* ( *col9a1c* ; accession number LC546948) coding sequence was amplified by PCR with primers (cloning_forward and cloning_reverse, see the Primer table for the sequence) that we previously described (Nakagawa et al., 2022) . Subsequently, an *EGFP* -inserted *col9a1c* construct was generated. Three PCR fragments­—(1) 5′end of *col9a1c* ­－V5 tag, (2) V5 tag－EGFP, and (3) EGFP－3′end of *col9a1c* —were amplified with the primer pairs described in the Primer table. Then these fragments were united using the In-Fusion HD cloning kit (TaKaRa). The construct was cloned into a pTol2- *and1mes* plasmid vector that has a fin mesenchymal cell specific promoter and an *IRES-Hsa.HIST1H2BJ-mRFP* reporter cassette (referred to as *IRES-H2BRFP* in Fig. 1A ). The *and1mes* promoter was set to 5 tandem repeats to enhance expression of the construct. The plasmid (final concentration of 10 ng/mL) was co-injected with *Tol2* transposase mRNA (final concentration of 25 ng/mL) into single-cell stage zebrafish (Tübingen) embryos. Transgene integration was confirmed by observing EGFP and RFP expression using a BZ-8000K fluorescent microscope (KEYENCE). The bright-field images at 5 dpf were obtained by BZ-X710 inverted microscope (KEYENCE). The transgenic zebrafish line ( *5×and1:EGFP-col9a1c-IRES-Hsa.HIST1H2BJ-mRFP* ) is named ou2046Tg.

**Immunofluorescence staining**

Zebrafish were anesthetized briefly to allow amputation of the half-caudal fin. After fin amputation, fish were returned to the tank and most fish recovered from the damage. In 5 days post-amputation, regenerating fins were fixed with 4% paraformaldehyde (PFA) in PBS and stained as described by Lalonde and Akimenko (2018) and Kuroda et al. (2018) with primary antibodies against type 2 collagen (Developmental Studies Hybridoma Bank; Ⅱ-Ⅱ6B3, at 1:50). On the next day, Alexa 594-conjugated mouse IgG antibodies (Invitrogen, at 1:100) were used as the secondary antibodies for 2 h at room temperature (RT). After staining, the samples were cryosectioned.

**Cryosections**

The samples that were amputated and immune-stained with antibodies against Col2 were washed with PBS and dehydrated in increasing concentrations of sucrose in PBS (5%, 10% and 15% for 1 h at RT respectively, and 20% for overnight at 4 ℃). Next day, they were placed in O.C.T. compound (Sakura) containing 20% sucrose (1:1) for 2 h at RT. Following the embedding of samples in O.C.T. compound, they were frozen in liquid nitrogen and then cryosectioned at 10 µm thickness using a Leica CM1850 cryostat. The section images were acquired using a LSM780 confocal microscope (Carl Zeiss) and were analyzed using ZEN (Carl Zeiss).

## Reagents

**Primer table**

**Table:** 

	primer	sequence (5' to 3')
1	cloning_forward	TTTGGCAAAGAATTGTCGACCACCATGGCTCCTCATTTCAGG
2	V5tag_rev	CGAGGAGAGGGTTAGGGATAGGCTTACCATCTAATCTGTTCAAATCCAGC
3	V5tag_fwd	CTAACCCTCTCCTCGGTCTCGATTCTACGATGGTGAGCAAGGGCG
4	EGFP_rev	TTTACCATCTTTGCTCTTGTACAGCTCGTCCAT
5	EGFP_fwd	AGCAAAGATGGTAAAGGCGACACGGTGTGCC
6	cloning_reverse	TTAGGGGGGGGGGGCGGCCGCTCATGGACCCTTGATGTT

Primer 1 and 2 were used to amplify the fragment (1) 5′ end of *col9a1c* ­－V5 tag. Primer 3 and 4 were used to amplify the fragment (2) V5 tag－EGFP. Primer 5 and 6 were used to amplify the fragment (3) EGFP－3′ end of *col9a1c.* Note that primer 1 and 6 were the same primers described in Nakagawa et al. (2022).
